# Immunophenotyping of Circulating T Helper Cells Argues for Multiple Functions and Plasticity of T Cells *In Vivo* in Humans - Possible Role in Asthma

**DOI:** 10.1371/journal.pone.0040012

**Published:** 2012-06-29

**Authors:** Carina Malmhäll, Apostolos Bossios, Madeleine Rådinger, Margareta Sjöstrand, You Lu, Bo Lundbäck, Jan Lötvall

**Affiliations:** Krefting Research Centre, Department of Internal Medicine and Clinical Nutrition, Institute of Medicine, Sahlgrenska Academy, University of Gothenburg, Gothenburg, Sweden; University of Cape Town, South Africa

## Abstract

**Background:**

The immune process driving eosinophilic and non-eosinophilic asthma is likely driven by different subsets of T helper (Th) cells. Recently, *in vitro* studies and animal studies suggest that Th cell subsets displays plasticity by changing their transcription factor or by expressing multiple transcription factors. Our aim was to determine whether individuals with asthma and elevated circulating eosinophils express signs of different regulatory immune mechanisms compared with asthmatics with low blood eosinophils and non-asthmatic control subjects. In addition, determine the relationship between eosinophilia and circulating Th cell subsets.

**Methodology/Principal findings:**

Participants were selected from a random epidemiological cohort, the West Sweden Asthma Study. Immunophenotypes of fresh peripheral blood cells obtained from stable asthmatics, with and without elevated eosinophilic inflammation (EOS high and EOS low respectively) and control subjects, were determined by flow cytometry. No differences in the number of Th1 (T-bet), Th2 (GATA-3), Th17 (RORγt) or Treg (FOXP3) cells were observed between the groups when analysing each subset separately. However, in all groups, each of the Th subsets showed expression of additional canonical transcription factors T-bet, GATA-3, RORγt and FOXP3. Furthermore, by *in vitro* stimulation with anti-CD3/anti-CD28 there was a significant increase of single expressing GATA-3^+^ and co-expressing T-bet^+^GATA-3^+^ cells in the EOS high asthmatics in comparison with control subjects. In addition, T-bet**^−^**GATA-3^+^RORγt^+^FOXP3^+^ were decreased in comparison to the EOS low asthmatics. Finally, in a group of control subjects we found that the majority of proliferating Th cells (CD4^+^CD25^+^Ki67^+^) expressed three or four transcription factors.

**Conclusions:**

The ability of human Th cells to express several regulatory transcription factors suggests that these cells may display plasticity *in vivo*.

## Introduction

Asthma is a heterogeneous disease; patients express different phenotypes and have differences in severity, natural history, and response to treatment [1]–[3]. It has been suggested that asthmatics can be divided into several subgroups, each with characteristic asthma symptoms that arise from distinct pathological processes [4]. A recent publication proposed that a subtype of asthma with a distinct underlying pathology can be classed as an “endotype” [5]. Cluster analysis of asthma phenotypes has identified distinct subgroups of patients, some of which have eosinophilic inflammation [2]. Phenotyping studies on these groups of asthmatics may shed some light on the immunological processes underlying these phenotypically-distinct disease subtypes and help to identify and characterise asthma endotypes.

In addition to the well characterised T regulatory (Tregs) cells and T helper (Th) cells, such as Th1 and Th2, new effector cells, including Th17, Th9 and Th22 have been identified in the last few years [6]. Until recently, Th effector cells were considered to be fully differentiated, with a permanent function upon expression of a characteristic transcription factor and their development from naïve CD4 cells was thought to be lineage specific [7]. However, there is accumulating evidence from animal models and *in vitro* studies to suggest that Th cell subsets are not irreversibly differentiated, but can exhibit plasticity by changing transcription factor expression or by expressing multiple transcription factors [8]–[12]. This plasticity of T cell differentiation has recently been suggested to play a role in modulating inflammatory disease [8], [13]. To date, this evidence has mainly come from experimental studies [14]–[18]; however, it raises the question of whether the same phenomenon occurs in circulating T cells in healthy humans and/or asthmatics.

Our hypothesis was that patients with a specific asthma endotype could be distinguished by the immunological profile of their circulating Th cells. Therefore, we compared the absolute numbers of circulating Th cells expressing the transcription factors, T-bet, GATA-3, RORγt and FOXP3, in two groups of asthmatics. All participants, both asthmatics and healthy subjects, were selected from an epidemiologically based asthma cohort study, the West Sweden Asthma Study. The asthmatics displayed similar clinical disease profiles, but with distinct differences in the number of circulating eosinophils, and were compared to healthy subjects.

## Materials and Methods

### Study Subjects

Study participants were selected from questionnaire respondents in the West Sweden Asthma Study (aged 16–75 years) who attended a detailed clinical examination at the Krefting Research Centre, Gothenburg, Sweden and for whom clinical data was available (Visit 1) [19]. Study participants attending Visit 1 and fulfilling inclusion criteria described below, were invited to participate in the study. Asthma was diagnosed from reports of common symptoms and a PD20 for methacholine below a cumulative dose of 1.94 µg or a FEV_1_ reversibility greater than 12%. The skin prick test (SPT) was performed using a standard panel of 11 inhalant allergens composed of birch, mugwort, timothy, horse, dog, cat, cockroach, *Cladosporium, Alternaria, Dermatophagoides farinae and D. pteronyssinus* (ALK, Hørsholm, Denmark). The SPT test was considered positive with a wheal and flare reaction ≥3mm for at least one allergen. Asthmatics were considered to have high numbers of eosinophils if blood eosinophils were ≥0.3×10^9^/L and low eosinophils if values were ≤0.2×10^9^/L. Control subjects did not report asthma symptoms, were non-reactive to methacholine or non-reversible, were SPT negative and had a low number of blood eosinophils. All participants were non-smokers.

The study population consisted of 11 asthmatics with high blood eosinophils (EOS high group), 12 asthmatics with low blood eosinophils (EOS low group) and 9 non-asthmatic healthy controls (control group). Participants were recruited in two separate blocks either during winter time (December to February) or during spring time (April to early June). During a clinical visit (Visit 2) blood samples, nasal lavage (NAL) and induced sputum were collected in addition to spirometry and fractional exhaled nitric oxide (FeNO) measurements were taken. During four weeks prior to Visit 2, none of the subjects had received any vaccination, changed their asthma medication, had any worsening of asthma symptoms, reported any symptoms of infection/cold, had any surgery, had any antibiotics, had any new medication, or had any anti-inflammatory medication (*i.e*. NSAIDs). The time period separating Visit 1 and Visit 2 ranged between 3 months to 2 years.

All subjects gave written informed consent. None of the participants of this study were under the age of eighteen. Ethical approval for the study was granted by the Regional Ethical Approval Committee in Gothenburg, Sweden (no. 593-08).

### Cells and Immunostaining

Peripheral whole blood cells were stained for flow cytometry within 1 hour of sampling. The total cell count was obtained using Trucount™ tubes (BD Biosciences, San Jose, CA, USA) together with antibodies (Ab) detecting CD45/CD3/CD4 or CD45/CD3/CD4/CD14/CD19 according to manufacturer’s protocol. Additionally, the following Ab panels were used: a) CD4/CD25/T-bet/GATA-3/RORγt/FOXP3 with or without CD3, b) CD45RO/CD45RA/CD3/T cell receptor (TCR)αβ/TCRγδ/CD4/CD8, c) CD45/CD56/CD16/CD19/CD14, d) CD4/CD25/Ki-67/T-bet/GATA-3/RORγt and e) CD4/CD25/Ki-67/T-bet/RORγt/FOXP3. All monoclonal antibodies were purchased from BD Biosciences, eBioscience or Invitrogen (detailed in Table S3). A Foxp3 Staining Buffer Set (eBioscience, Inc. San Diego, CA, USA) was used according to manufacturer’s protocol for all intracellular staining.

Human peripheral blood mononuclear cells (PBMCs) were isolated by density centrifugation using Ficoll-Paque™ (GE Healthcare Bio-Sciences, Uppsala, Sweden) and cultured in serum-free AIM-V® + AlbuMAX® medium (Invitrogen, GIBCO, Grand Island, NY, USA) alone or in plates pre-coated with anti-CD3/anti-CD28 antibodies (1 µg each/mL; BD Pharmingen™, BD Biosciences) for 48 hours, before flow cytometry staining with antibody panel A (see above). Cell viability was analysed using 7AAD exclusion in the forward scatter-side scatter (FSC-SSC) gate. Cell viability was >96% (range 96.3–99.1%) in cells harvested from medium alone and >88% (range 88.4–95.8%) in cells harvested from anti-CD3/anti-CD28 coated wells.

### Flow Cytometry

Samples were processed on a BD FACS Aria (BD Biosciences, San Jose, CA, USA) and data were analyzed with FlowJo software (Tree Star, Ashland, OR). Gating of transcription factors and surface markers were determined using control samples by the Fluorescence Minus One (FMO) approach *i.e*. controls containing all markers except the one of interest were used to set gates (Figure S1).

### Cytospin Preparations and Microscopy

Cells from induced sputum and NAL were stained with May-Grünwald and Giemsa (HistoLab Products AB, Gothenburg, Sweden) and counted using a Zeiss Axioplan microscope (Carl Zeiss Jena GmbH, Eching, Germany; Methods S1).

CD4 T lymphocytes were enriched from PBMCs according to the manufacturer’s protocol (Human CD4 T Lymphocyte Enrichment Set, BD IMag™, BD Pharmingen™, BD Biosciences) and stained with the following combinations: a) T-bet/GATA-3, b) FOXP3/GATA-3, c) T-bet/RORγt and d) FOXP3/RORγt. The detection signal was enhanced using fluorochrome-conjugated secondary antibodies (Table S3). Cytospins were made after completion of the staining procedure and mounted with ProLong® Gold antifade reagent with DAPI (Invitrogen™, Molecular Probes®, Eugene, OR, USA). Images were captured with a Zeiss LSM 510 META microscope using Zeiss LSM 510 software (Carl Zeiss MicroImaging GmbH, Germany). Confocal images are made by Z sectioning at scale 0.38 µm. Technical controls are shown in figure S2.

### Statistical Analyses

Data were tested for adherence to a normal distribution with the Kolmogorov-Smirnov test (with Dallal-Wilkinsson-Lilliefor corrected P value). If a normal distribution of sample means could be assumed, unpaired test or paired t-test was used; otherwise the Kruskal-Wallis test followed by the Mann-Whitney U test were applied. Values in tables and graphs are mean±SEM, except in scatter plot graphs where median values are given. Correlations were performed using Pearson correlation test. Values of p<0.05 were considered statistically significant.

## Results

### Eosinophilia

Clinical characteristics and standard hospital laboratory measurements were similar between the study groups, except for a higher number of eosinophils in the blood in the EOS high group compared with the EOS low and control groups (p<0.001; Table 1). No differences were observed between the groups in either the numbers of any general T cell population, CD3/CD4/CD8, their memory/naïve subpopulations or TCRγδ cells. However, TCRαβ cells showed increased numbers in the EOS high group compared to the EOS low group of asthmatics. Furthermore, the number of monocytes, natural killer (NK) and B cells were similar between the three groups (Table S1). The relative number of eosinophils in induced sputum was significantly higher in the EOS high group compared with the healthy controls (p<0.05) and higher in comparison to the EOS low group, although the difference was not significant (Table 1).

**Table 1:** Demographic and clinical characteristics of study participants.

Parameter	Units	Healthy controls		Asthmatics EOS High		Asthmatics EOS Low	
		mean (±SEM)		mean (±SEM)		mean (±SEM)	
No. of participants	M/F	6/3		4/7		6/6	
		Visit 1	Visit 2	Visit 1	Visit 2	Visit 1	Visit 2
Age	years	37.9 (3.6)	39.0 (3.6)	38.7 (3.4)	40.1 (3.4)	39.8 (2.5)	40.9 (2.5)
BMI		24.2 (0.7)		26.3 (1.8)		27.3 (1.6)	
FeNO	ppb	15.9 (1.8); n = 7	16.8 (2.2)	48.4 (13.6); n = 9	28.1 (6.6)	23.8 (3.8)	22.2 (4.8)
FEV_1_, predicted	%	102.1 (4.4)	94.5 (3.1); n = 8	99.4 (3.1)	94.2 (4.1)	94.7 (3.7)	88.4 (4.6); n = 11
FEV_1_ reversibility	%	6.3 (1.2)		12.6 (4.5); n = 10		7.5 (2.1)¶¶	
Methacholine, PD20	µg	¶		268.8 (126.7)		613.7 (131.5); n = 11	
C-reactive protein	mg/L		3.0 (1.9)		2.3 (0.8)		1.4 (0.6)
Blood eosinophils	x10^9^/L	0.12 (0.02)	0.13 (0.02)	0.37 (0.03)†***	0.32 (0.03)†***	0.10 (0.01)	0.14 (0.02)
Blood neutrophils	x10^9^/L	3.26 (0.25)	3.78 (0.42)	3.36 (0.28)	3.48 (0.31)	3.35 (0.28)	3.42 (0.34)
Blood basophils	x10^9^/L	0.01 (0.01)	0.01 (0.01)	0.05 (0.02)	0.04 (0.02)	0.02 (0.01)	0.01 (0.01)
Blood monocytes	x10^9^/L	0.38 (0.05)	0.39 (0.05)	0.47 (0.04)	0.40 (0.05)	0.40 (0.03)	0.35 (0.03)
Blood lymphocytes	x10^9^/L	1.94 (0.13)	1.98 (0.20)	1.99 (0.12)	2.27 (0.15)	1.82 (0.09)	2.04 (0.17)
Sputum eosinophils	% of total cells		0.70 (0.35); n = 8		7.62 (2.89)††*		1.80 (0.58); n = 11
NAL eosinophils	% of total cells		0.96 (0.40); n = 8		3.87 (1.45); n = 10		1.54 (1.01)
Skin prick test	+/−	0/9		8/3		9/3	
Medication							
Inhaled steroids¶¶¶	Yes	0	0	4	4	5	1
	No	9	9	3	4	4	9
	Intermittently	0	0	3	3	3	2
β_2_-agonist¶¶¶¶	Yes	0	0	3	2	0	1
	No	9	9	1	1	6	6
	Intermittently	0	0	7	8	6	5
Combination medication¶¶¶¶¶	Yes	0	0	1	1	0	0
	No	9	9	9	8	10	10
	Intermittently	0	0	1	2	2	2

¶ Non-reactive to methacholine up to a dose of 1.96 mg.

¶¶ One individual, not subjected to methacholine, was selected based on FEV_1_ reversability with a value of 25%.

¶¶¶ Budesonide 400 µg/day at visit 2for all users, except one individual in EOS low group with a usage of 200 µg/day.

¶¶¶¶ Short acting β_2_-agonist is most frequently used.

¶¶¶¶¶ Budesonide 320 µg/day at visit 2 for all users.

† Statistically significant in comparison with EOS low and healthy controls (***P<0.001).

†† Statistically significant in comparison with healthy controls (*P<0.05).

### Th Cell Transcription Factors

**Figure 1:**
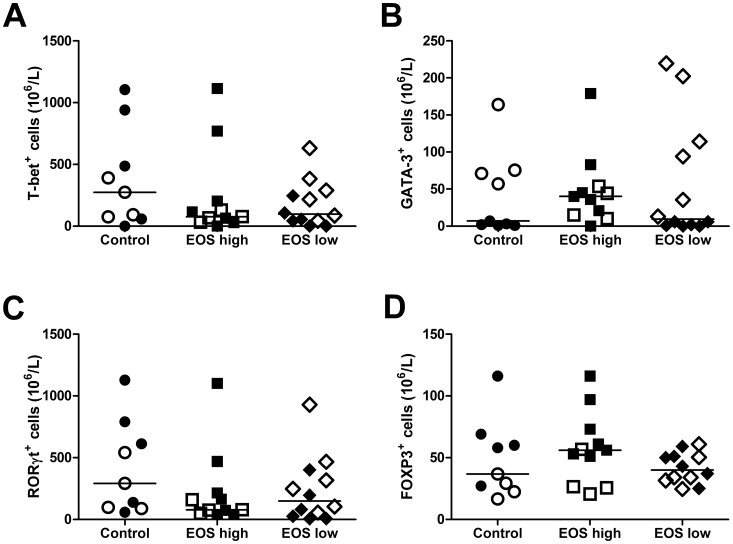
Transcription factors expressed by T cell subtypes. Numbers of Th subtypes expressing the transcription factors T-bet (Th1; A), GATA-3 (Th2; B) RORγt (Th17; C) and FOXP3 (Treg; D) in the control (healthy non-asthmatics, n = 9), EOS high (asthmatics with elevated blood eosinophils, n = 11), and EOS low (asthmatics with low blood eosinophils, n = 12) groups. The horizontal line represents the median of the group. Filled symbols represent samples obtained in the winter time, while open symbols represent samples obtained during spring time.

The number of CD4^+^CD25^+^ cells and the expression of the transcription factors T-bet, GATA-3, RORγt and FOXP3 was analysed in the groups. The total number of CD4^+^CD25^+^ T cells did not differ between the groups (Table S1). The levels of transcription factors are presented in Figure 1. There were no differences in the numbers of T-bet^+^, GATA-3^+^, RORγt^+^ or FOXP3^+^ cells between the groups when analysing each subset separately (Figure 1A-D).

### Analysis of Th Cells Expressing Multiple Master Regulatory Transcription Factors

**Figure 2:**
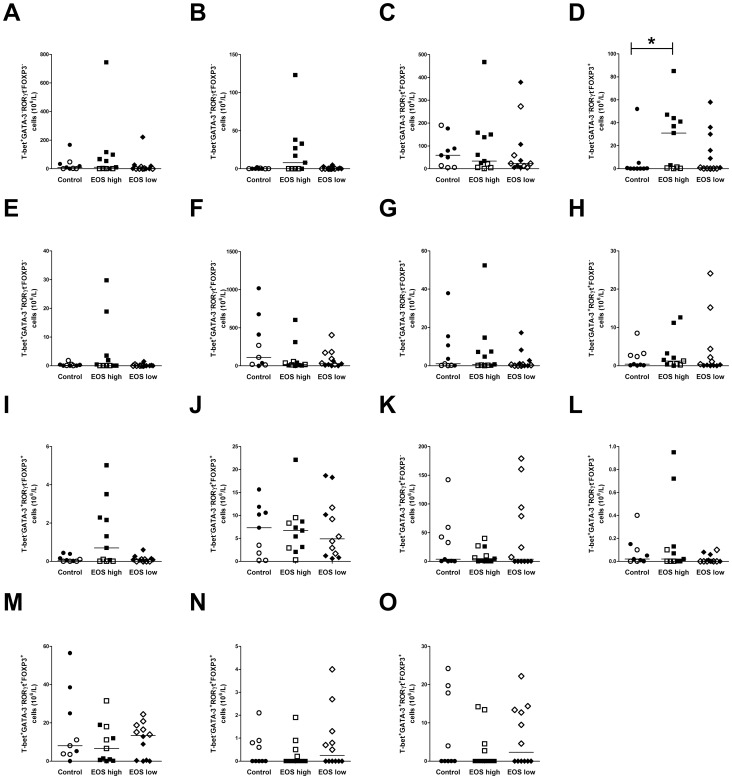
Sub analysis of circulating T-helper cell subtypes. Numbers of each subtype in the control (healthy non-asthmatics, n = 9), EOS high (asthmatics with elevated blood eosinophils, n = 11), and EOS low (asthmatics with low blood eosinophils, n = 12) groups. CD4^+^CD25^+^ cells positive for only one of the transcription factors T-bet^+^ (A), GATA-3^+^ (B), RORγt^+^ (C) and FOXP3^+^ (D). CD4^+^CD25^+^ cells co-expressing two transcription factors T-bet^+^GATA-3^+^ (E), T-bet^+^RORγt^+^ (F), T-bet^+^FOXP3^+^ (G), GATA-3^+^RORγt^+^ (H), GATA-3^+^FOXP3^+^ (I) and RORγt^+^FOXP3^+^ (J). CD4^+^CD25^+^ cells positive for three transcription factors T-bet^+^GATA-3^+^RORγt^+^ (K), T-bet^+^GATA-3^+^FOXP3^+^ (L), T-bet^+^RORγt^+^FOXP3^+^ (M) and GATA-3^+^RORγt^+^FOXP3^+^ (N). CD4^+^CD25^+^ cells expressing all four transcription factors T-bet^+^GATA-3^+^RORγt^+^FOXP3^+^ (O). The horizontal line represents the median of the group. *p<0.05. Filled symbols represent samples obtained in the winter time, while open symbols represent samples obtained during spring time.

In addition to quantifying the different Th cell subsets, the number of cells co-expressing the transcription factors T-bet, GATA-3, RORγt and FOXP3 as measured in each subset of Th cells (Figure 2). Cells expressing only FOXP3 were increased in the EOS high group compared with healthy controls (p<0.05; Figure 2 D). In some cell subtypes, up to four transcription factors were co-expressed but none of the co-expressing subtypes showed any significant difference between groups.

**Figure 3:**
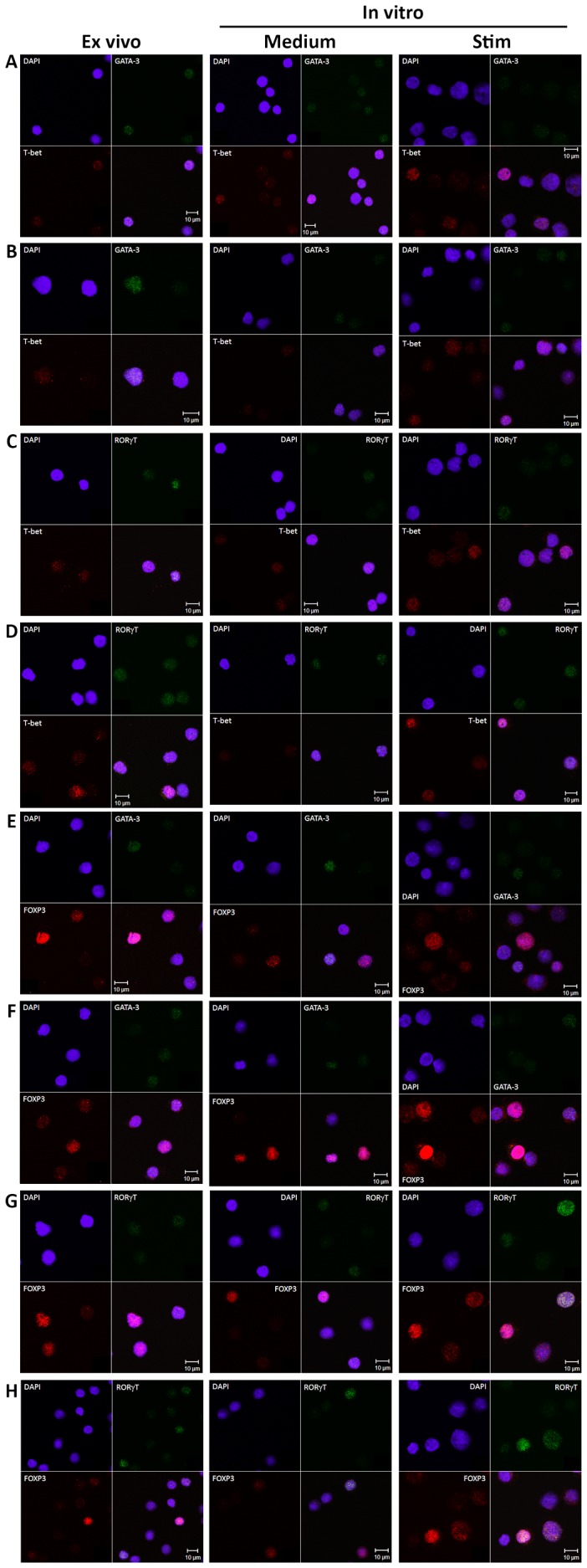
Confocal microscopy showing co-expression of transcription factors. The three columns represent immunostained cells from fresh *ex vivo* CD4-enriched cells (1^st^ column), CD4-enriched cells *in vitro* in medium alone (2^nd^ column) or after stimulation with anti-CD3/anti-CD28 antibodies (3^rd^ column) for 48 hours respectively. Row A, C, E and G are samples from one non-asthmatic control subject. Row B, D, F and H are sample from an asthmatic individual with elevated blood eosinophils. Cells were stained with DAPI (blue nuclear staining) and transcription factors T-bet (Th1), GATA-3 (Th2), RORγt (Th17) and FOXP3 (Treg). A+B shows co-expression of T-bet/GATA-3; C+D, T-bet/RORγt; E+F, FOXP3/GATA-3; G+H, FOXP3/RORγt.

To confirm the flow cytometry data of co-expression of transcription factors in CD4^+^ cells, confocal microscopy was performed. CD4^+^ cells from the peripheral blood of a control subject and an asthmatic individual with elevated blood eosinophils were enriched and immunostained using the same clones of primary antibodies used for flow cytometric staining, but with the addition of fluorochrome conjugated secondary antibodies. This technique enabled the visualisation of the expression of two transcription factors in the same sample, specifically, T-bet/GATA-3 (Figure 3A+B, *ex vivo*), T-bet/RORγt (Figure 3C+D, *ex vivo*), FOXP3/GATA-3 (Figure 3E+F, *ex vivo*) and FOXP3/RORγt (Figure 3G+H, *ex vivo*).

### *In vitro* Stimulation Increases the Number of Cells Expressing Multiple Transcription Factors

**Figure 4:**
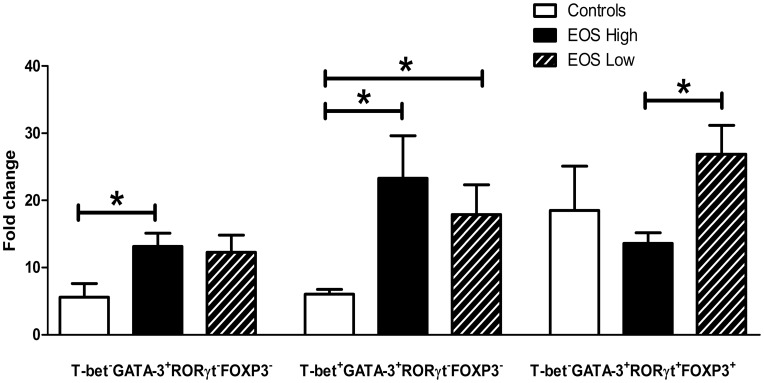
Th cells increase after *in vitro* TCR stimulation. Three subsets of Th cells displayed fold change differences between the study groups: single expressing GATA-3^+^, co-expressingT-bet^+^GATA-3^+^ and GATA-3^+^RORγt^+^FOXP3^+^, after 48 hours *in vitro* stimulation with anti-CD3/anti-CD28 antibodies. Data are expressed as mean±SEM. *p<0.05.

To determine if *in vitro* stimulation of the cells could change the co-expression pattern of transcription factors, PBMCs taken from all groups during winter time were cultured with a combination of anti-CD3 and anti-CD28 antibodies. After 48 hours, cells were harvested and stained for co-expression of T-bet, GATA-3, RORγt and FOXP3. The number of cells expressing multiple and single transcription factors was expressed as a percentage of the total CD4^+^ T cell count and the effect of stimulation compared with medium alone. A sub analysis showed that most Th subsets increased after stimulation compared to medium alone but not all (Table 2). Single FOXP3^+^ and T-bet^+^FOXP3^+^ cells were not increased in any group. Single T-bet^+^ and GATA-3^+^FOXP3^+^ cells were only increased in EOS high group. RORγt^+^FOXP3^+^ cells were only increased in the asthmatic groups. To compare between groups, the fold change was calculated by dividing the number of cells obtained from stimulated samples by the number of cells obtained from medium alone (Table S2). Three subtypes of cells differed between the groups; single GATA-3^+^ cells were increased in the EOS high group in comparison to controls, T-bet^+^GATA-3^+^ cells were increased in both asthmatic groups and GATA-3^+^RORγt^+^FOXP3^+^ cells were decreased in the EOS high group compared to the EOS low group (Figure 4). Non-specific cell stimulation without the addition of exogenous polarising cytokines can thus increase the number of cells co-expressing multiple master regulatory transcription factors in comparison to non-stimulated cells, indicating that this is an active process. This co-expression data was confirmed by repeating the confocal microscopy on CD4^+^ cells enriched from cultured PBMCs as previously mentioned above. Micrographs showed co-expression of T-bet/GATA-3 (Figure 3A+B, *in vitro*), T-bet/RORγt (Figure 3C+D, *in vitro*), and FOXP3/GATA-3 (Figure 3E+F, *in vitro*) and FOXP3/RORγt (Figure 3G+H, *in vitro*) in CD4^+^ cells both from stimulated cells as well as in medium alone treated cells. However, in the medium alone treated samples, co-expressing cells were fewer overall than in the stimulated samples.

**Table 2:** The expression of *in vitro* transcription factors.

Parameter	Healthy controls		Asthmatics EOS High		Asthmatics EOS Low	
	Mean (±SEM)		Mean (±SEM)		Mean (±SEM)	
	n = 5		n = 7		n = 6	
	Medium	Stim.	Medium	Stim.	Medium	Stim.
**T-bet^+^** GATA-3**^−^** RORγt**^−^** FOXP3**^−^**	0.13 (0.03)	0.23 (0.04)	0.11 (0.03)	0.20 (0.04)*	0.10 (0.02)	0.28 (0.09)
T-bet**^−^** **GATA-3^+^** RORγt**^−^** FOXP3**^−^**	0.03 (0.01)	0.15 (0.04)*	0.01 (0.00)	0.17 (0.02)***	0.02 (0.00)	0.18 (0.02)**
T-bet**^−^** GATA-3**^−^** **RORγt^+^** FOXP3**^−^**	0.69 (0.16)	6.06 (1.57)*	1.02 (0.25)	5.58 (0.80)**	0.54 (0.10)	5.43 (0.81)**
T-bet**^−^** GATA-3**^−^** RORγt**^−^ FOXP3^+^**	0.49 (0.10)	0.49 (0.12)	0.63 (0.15)	0.43 (0.05)	0.70 (0.16)	0.40 (0.06)
**T-bet^+^ GATA-3^+^** RORγt**^−^** FOXP3**^−^**	0.01 (0.00)	0.06 (0.01)**	0.00 (0.00)	0.08 (0.02)**	0.00 (0.00)	0.08 (0.02)**
**T-bet^+^** GATA-3**^−^** **RORγt^+^** FOXP3**^−^**	1.21 (0.21)	25.6 (3.66)**	1.79 (0.42)	16.78 (2.42)**	1.14 (0.16)	19.41 (2.76)**
**T-bet^+^** GATA-3**^−^** RORγt**^−^ FOXP3^+^**	0.11 (0.06)	0.10 (0.04)	0.13 (0.07)	0.07 (0.02)	0.13 (0.05)	0.08 (0.02)
T-bet**^−^** **GATA-3^+^** **RORγt^+^** FOXP3**^−^**	0.09 (0.03)	2.19 (0.43)*	0.12 (0.04)	3.30 (0.57)**	0.07 (0.02)	2.66 (0.34)**
T-bet**^−^** **GATA-3^+^** RORγt**^−^ FOXP3^+^**	0.02 (0.00)	0.07 (0.02)	0.03 (0.01)	0.07 (0.01)**	0.03 (0.01)	0.06 (0.01)
T-bet**^−^** GATA-3**^−^** **RORγt^+^ FOXP3^+^**	2.04 (0.62)	3.52 (0.55)	1.55 (0.28)	3.39 (0.51)**	1.20 (0.18)	3.28 (0.48)*
**T-bet^+^ GATA-3^+^ RORγt^+^** FOXP3**^−^**	0.37 (0.14)	21.24 (3.86)**	0.38 (0.16)	30.29 (2.82)***	0.25 (0.08)	27.74 (3.06)***
**T-bet^+^ GATA-3^+^** RORγt**^−^ FOXP3^+^**	0.01 (0.00)	0.02 (0.01)*	0.01 (0.00)	0.02 (0.00)	0.01 (0.00)	0.02 (0.00)
**T-bet^+^** GATA-3**^−^** **RORγt^+^ FOXP3^+^**	2.74 (0.76)	17.48 (2.89)**	2.32 (0.40)	14.57 (3.16)*	2.10 (0.33)	16.06 (2.57)**
T-bet**^−^** **GATA-3^+^ RORγt^+^ FOXP3^+^**	0.11 (0.04)	1.20 (0.24)*	0.12 (0.02)	1.57 (0.22)***	0.06 (0.01)	1.49 (0.19)**
**T-bet^+^ GATA-3^+^ RORγt^+^ FOXP3^+^**	0.19 (0.03)	8.32 (1.83)*	0.23 (0.05)	12.95 (2.52)**	0.17 (0.06)	11.79 (1.98)**

Values are expressed as percent of CD4^+^ T cells.

* P<0.05.

** P<0.01.

*** P<0.001 in comparison to medium alone.

Stim.  =  stimulated. Medium  =  medium alone.

Bold lettering highlights the transcription factor of interest.

### Proliferating Th Cells Express Multiple Transcription Factors

**Figure 5:**
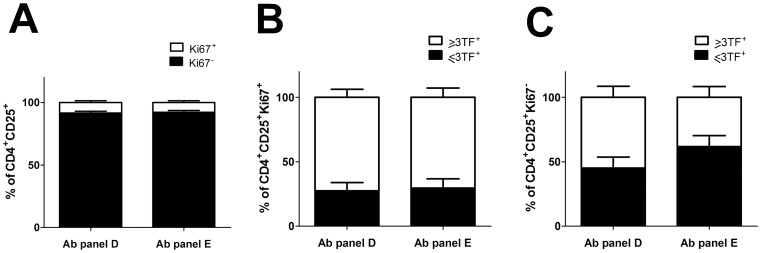
Proliferating CD4^+^CD25^+^ cells expressing multiple transcription factors in fresh *ex vivo* whole blood from healthy non-asthmatic controls (n = 5). Antibody panel D: stained simultaneously with Ki-67/T-bet/GATA-3/RORγt. Antibody panel E: Ki-67/T-bet/RORγt/FOXP3. A) Percentage of Ki-67^+^ (open bar) and Ki-67**^−^** (filled bar) CD4^+^CD25^+^ cells. B) Percentage of CD4^+^CD25^+^Ki-67^+^ expressing ≥ three transcription factors (open bar) or ≤three transcription factors (filled bar). C) Percentage of CD4^+^CD25^+^Ki-67**^−^** expressing ≥ three transcription factors (open bar) or ≤three transcription factors (filled bar). Data are expressed as mean±SEM.

Data on subsets of Th cells in circulation that are proliferating are shown in figure 5. Proliferating cells are cells positive of the marker Ki-67. Two different Ab panels, Panel D (CD4/CD25/Ki-67/T-bet/GATA-3/RORγt) and Panel E (CD4/CD25/Ki-67/T-bet/RORγt/FOXP3) were used, since only three different transcription factors in addition to Ki-67 could be evaluated simultaneously. Therefore, we present the data as more or equal to three transcription factors (≥3TFs) in figure 5B and 5C. In a group of healthy controls (n = 5) we found that 8.44% of CD4^+^CD25^+^ cells in Panel D and 7.44% of CD4^+^CD25^+^ cells in Panel E comprise of Ki-67^+^ cells (Figure 5A). Most of the Ki-67^+^ cells were co-expressing the three transcriptions factors in each of the Ab panels (Panel D 72.44±6.21%; Panel E 70.41±7.29%; Figure 5B). On the contrary, we found that in the Ki-67**^−^** fraction (Figure 5C), there were more cells positive for T-bet/GATA-3/RORγt (Panel D; 48.19±7.95%) than cells positive for T-bet/RORγt/FOXP3 (Panel E; 31.88±7.01%). Taken together, these data suggests that Th cells co-expressing multiple transcription factors can be both actively proliferating or resting cells.

### Relationship between Th Cell Subsets and Clinical Parameters in Asthmatics

To evaluate the possible clinical importance of the subsets analysed, both single expressing and co-expressing Th cells were compared to clinical parameters in the asthmatic subjects, using a Pearson correlation test. Each subset was tested against the main clinical asthma characteristics including FeNO, FEV_1%_ predicted_,_ FEV_1_ reversibility, and sputum-eosinophils. The highest correlation score (R = 0.760; p<0.0005) between any cell subset and any of the clinical parameters was single expressing GATA-3^+^ cells versus FEV_1_ reversibility. In addition, two more GATA-3 subsets, T-bet^+^GATA-3^+^ and T-bet^+^GATA-3^+^FOXP3^+^, showed correlation to FEV_1_ reversibility (Table 3). Furthermore, FeNO correlated to single expressing RORγt in addition to two additional RORγt subsets, T-bet^+^RORγt^+^ and RORγt^+^FOXP3^+^ cells (Table 3). However, when performing the correlation test using the subgroups of asthmatics EOS high and EOS low, only five of the subsets in the EOS high group showed significant correlation to FeNO or FEV_1_ reversibility and none in the EOS low group (Table 3), which implies that GATA-3 and RORγt expression may have clinical relevance in asthma subgroups.

**Table 3:** Relationship between Th cell subsets and clinical parameters in asthmatics.

Asthmatics				
		Pearson correlation(R value)	P value	No. ofparticipants
FeNO	T-bet**^−^** GATA-3**^−^** **RORγt^+^** FOXP3**^−^**	0.478	0.021	23
	**T-bet^+^** GATA-3**^−^** **RORγt^+^** FOXP3**^−^**	0.478	0.021	23
	T-bet**^−^** GATA-3**^−^** **RORγt^+^ FOXP3^+^**	0.482	0.020	23
FEV_1_% reversibility	T-bet**^−^** **GATA-3^+^** RORγt**^−^** FOXP3**^−^**	0.760	0.0005	22
	**T-bet^+^ GATA-3^+^** RORγt**^−^** FOXP3**^−^**	0.591	0.004	22
	**T-bet^+^ GATA-3^+^** RORγt**^−^ FOXP3^+^**	0.554	0.007	22
**EOS high asthmatics**				
FeNO	T-bet**^−^** GATA-3**^−^** **RORγt^+^** FOXP3**^−^**	0.763	0.006	11
	**T-bet^+^** GATA-3**^−^** **RORγt^+^** FOXP3**^−^**	0.711	0.014	11
	T-bet**^−^** GATA-3**^−^** **RORγt^+^ FOXP3^+^**	0.669	0.024	11
FEV_1_% reversibility	T-bet**^−^** **GATA-3^+^** RORγt**^−^** FOXP3**^−^**	0.868	0.001	10
	**T-bet^+^ GATA-3^+^** RORγt**^−^** FOXP3**^−^**	0.651	0.042	10
	**T-bet^+^ GATA-3^+^** RORγt**^−^ FOXP3^+^**	0.599	ns	10
**EOS low asthmatics**				
FeNO	T-bet**^−^** GATA-3**^−^** **RORγt^+^** FOXP3**^−^**	0.084	ns	12
	**T-bet^+^** GATA-3**^−^** **RORγt^+^** FOXP3**^−^**	0.032	ns	12
	T-bet**^−^** GATA-3**^−^** **RORγt^+^ FOXP3^+^**	0.317	ns	12
FEV_1_% reversibility	T-bet**^−^** **GATA-3^+^** RORγt**^−^** FOXP3**^−^**	−0.295	ns	12
	**T-bet^+^ GATA-3^+^** RORγt**^−^** FOXP3**^−^**	−0.493	ns	12
	**T-bet^+^ GATA-3^+^** RORγt**^−^ FOXP3^+^**	−0.036	ns	12

Bold lettering highlights the transcription factor of interest.

## Discussion

This study shows for the first time, using flow cytometry and confocal microscopy, that circulating Th cells can be classified into at least two main categories; those that express only one of the master regulatory transcription factors: T-bet, GATA-3, FOXP3, or RORγt or those that co-express two or more of these transcription factors. These findings indicate that many circulating Th cells, in both asthmatics and healthy controls; express multiple transcription factors which support the concept that individual Th cells may have multiple functions *in vivo* in humans. However, from the *in vitro* data, it was shown that only single expressing or multi expressing GATA-3 cells were upregulated in asthmatics compared to controls upon stimulation.

Until recently, Th cells expressing a master regulatory transcription factor were considered to be lineage-committed cells with particular characteristics, including the release of defined cytokines. This dogma has recently been questioned, by several studies that suggest T cells can exhibit functional plasticity *in vitro* [8]–[12]. Furthermore, it is likely that the multi-transcription factor Th cells are not naïve, as the present study shows that they express CD25, arguing that the cells have been activated. Although the possibility of recent cell activation cannot be excluded [20], the participants recruited into the study were not undergoing any asthma exacerbations, and their symptoms were stable.

Sub-analysis of the T-bet, GATA-3, FOXP3, and RORγt cell populations, enabled up to 15 different populations to be defined. These populations expressed single transcription factors or up to four transcription factors, arguing for the presence of Th cell plasticity in humans as has been earlier proposed from animal models or *in vitro* [14]–[18]. The strength of this current study is that these cells were detected in fresh blood obtained within one hour of sampling, without any form of artificial manipulation. To date, this is the first study showing that circulating Th cells co-express several transcriptions factors, which are considered to function as master regulators. These results raise interesting questions like; what is the function of these co-expressing Th cells, and which transcription factor is dominant? These questions were beyond the scope of this study, but data from other *in vitro* studies suggest that they may have multiple functions. Voo et al recently showed that FOXP3^+^ interleukin (IL)-17^+^ cells expressing RORγt still showed suppressive function *in vitro* [21]. Furthermore, animal studies have shown that T-bet induction in Treg cells, after infection, facilitated these cells to supress Th1 inflammation [22], [23]. Furthermore, in a model of non-allergic chronic lung inflammation, lung tissue CD4^+^IL-17^+^IL-13^+^IL-4^+^ cells co-expressed RORγt and GATA-3 after local or systemic immunization with inflammatory dendritic cells [24].

The only subset that was different after sub-analysis was single FOXP3 cells, which was increased only in EOS high asthmatics compared to controls. Increased Tregs in asthmatics has previously been shown [25]. On the contrary, there are also studies [26], [27] observing decreased FOXP3 expression in asthmatics. However, these investigators looked at a different cell population as they estimated the CD4^+^CD25^high^ cells that expressed FOXP3 and did not address the co-expression with others transcription factors [26], [27].

**Figure 6:**
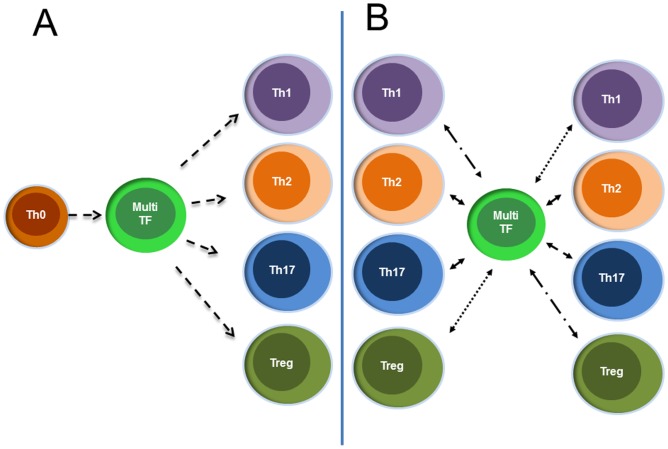
Alternative models for multiple transcription factor Th cells (Multi-TF). A) Multiple transcription factor cells are between naïve and terminally differentiated cells. B) Multiple transcription factor cells are differentiated cells that are transitioning from one cell type to another, going through a stage of multi expression before reaching the new type.

After 48 hours of non-specific CD3/CD28-activation *in vitro*, a substantial increase in the number of Th cells expressing multiple transcription factors was observed. Moreover, only a small percentage of stimulated cells were found to express a single transcription factor. This strongly argues that circulating Th cells, expressing a master regulatory transcription factor such as GATA-3 also have the capacity to express additional transcription factors upon further stimulation. The question remains unanswered, of whether Th cells that co-express multiple transcription factors are undergoing lineage commitment, or whether an already committed cell can express multiple master transcription factors (Figure 6). Several studies have investigated the co-expression of other key regulatory transcription factors in FOXP3^+^ Tregs. GATA-3 expression in Tregs is proposed to be a default response to TCR stimulation under neutral conditions, while under inflammatory settings, GATA-3 expression in Tregs limits the expression of transcription factors associated with T cell differentiation [17]. The microenvironment may have an important role for the balance of co-expression e.g T cells receiving a TGF-β signal can acquire the potential to develop into either Tregs or Th17 cells. In the absence of pro-inflammatory cytokines FOXP3 inhibits RORγt, but in the presence of these cytokines the opposite occur [18].

As *in vitro* studies showing that progenitor cells unable to proliferate remain uncommitted and bipotent, expressing both T-bet and GATA-3 mRNA in response to polarising signals for maturation [28] this study combined the transcription factor staining with a marker for proliferation, Ki-67. Interestingly, the data shows that actively proliferating cells co-express multiple transcription factors *ex vivo* to a great extent, at least in healthy control subjects. Furthermore, the present study is the first to confirm this co-expression of multiple transcription factors at the protein and single cell level in humans.

The population of this study was selected based on the number of circulating eosinophils, as the scope was to evaluate several transcription factors in an eosinophilic asthma phenotype. However, patients with elevated eosinophils within the normal range were chosen, as the aim was to study stable mild/moderate asthmatics without a “heavy” Th2 polarisation. It was surprising that no differences were detected in the total number of GATA-3 expressing CD4 cells, as this is considered the master transcription factor most related to eosinophilia [29]. However, upon TCR stimulation, only asthmatics showed upregulated GATA-3 cell subsets, arguing for a shift in this transcription factor. Furthermore, when a correlation test was applied between transcription factors and major clinical characteristics, the strongest correlations were found between GATA-3 and FEV_1_ reversibility, a key characteristic of asthma.

This study contains a relatively low number of asthmatics who were in a stable phase of disease, which may affect the results. Although beyond the scope of this study, analysis of single transcription factor and multi-transcription factor cells resident in the tissue of both control subjects, as well as asthmatics, would be of great interest in understanding the role in disease progression.

In summary, it has been demonstrated for the first time, that in both healthy individuals and individuals with mild/moderate stable asthma there are circulating Th cells expressing several transcription factors. This data argues against the key importance of a single transcription factor in stable asthma patients. However, TCR stimulation increased the GATA-3 cell populations, most profoundly in asthmatics. Furthermore, GATA-3 expression in fresh samples was correlated with FEV_1_ reversibility, a main feature of asthma.

## Supporting Information

Figure S1 **Flow cytometry analysis of Th cell subsets.** Representative FACS contour plots (level 10%) showing CD4^+^CD25^+^ cells gated for T-bet (Th1), GATA-3 (Th2), RORγt (Th17) and FOXP3 (Treg). One individual from each group is presented in the top three rows. In the final row, the fluorescence minus one (FMO) control used for analyses is shown.
(TIF)

Figure S2 **Technical controls for confocal microscopy.** Z sectioning is not performed on the following samples. Micrographs of *ex vivo* samples: 1) Only DAPI staining; 2) DAPI/mIgG1isotype control (IC)/Goat anti-mouse IgG-AF555; 3) DAPI/ratIgG2a IC/Goat anti-rat IgG-AF488; 4) DAPI/ratIgG2b IC/Goat anti-rat IgG-AF488; 5) DAPI/No primary antibody/Goat anti-mouse IgG-AF555/Goat anti-rat IgG-AF488; 6) DAPI/mouse anti-human FOXP3/Goat anti-mouse IgG-AF555; 7) DAPI/mouse anti-human T-bet/Goat anti-mouse IgG-AF555; 8) DAPI/Rat anti-human GATA-3/Goat anti-rat IgG-AF488; 9) DAPI/Rat anti-human RORγt/Goat anti-rat IgG-AF488; Micrographs of *in vitro* samples: 10) DAPI/mIgG1IC/Goat anti-mouse IgG-AF555 (medium alone); 11) DAPI/mIgG1IC/Goat anti-mouse IgG-AF555 (stimulated); 12) DAPI/ratIgG2a IC/Goat anti-rat IgG-AF488 (medium); 13) DAPI/ratIgG2a IC/Goat anti-rat IgG-AF488 (stim.); 14) DAPI/ratIgG2b IC/Goat anti-rat IgG-AF488 (medium); 15) DAPI/ratIgG2b IC/Goat anti-rat IgG-AF488 (stim.); 16) DAPI/No primary antibody/Goat anti-mouse IgG-AF555/Goat anti-rat IgG-AF488 (medium); 17) DAPI/No primary antibody/Goat anti-mouse IgG-AF555/Goat anti-rat IgG-AF488 (stim.); 18) DAPI/mouse anti-human FOXP3/Goat anti-mouse IgG-AF555 (medium); 19) DAPI/mouse anti-human FOXP3/Goat anti-mouse IgG-AF555 (stim.); 20) DAPI/mouse anti-human T-bet/Goat anti-mouse IgG-AF555 (medium); 21) DAPI/mouse anti-human T-bet/Goat anti-mouse IgG-AF555 (stim.); 22) DAPI/Rat anti-human GATA-3/Goat anti-rat IgG-AF488 (medium); 23) DAPI/Rat anti-human GATA-3/Goat anti-rat IgG-AF488 (stim.); 24) DAPI/Rat anti-human RORγt/Goat anti-rat IgG-AF488 (medium); 25) DAPI/Rat anti-human RORγt/Goat anti-rat IgG-AF488 (stim.).
(TIF)

Table S1 **Experimental laboratory data.**
(DOCX)

Table S2 **Fold change expression of** ***in vitro*** **transcription factors.**
(DOCX)

Table S3 **Antibodies used in the study.**
(DOC)

Methods S1 **Sputum induction and processing.**
(DOC)
