# Supplementary figures and images for: Immunophenotyping of Circulating T Helper Cells Argues for Multiple Functions and Plasticity of T Cells In Vivo in Humans - Possible Role in Asthma
Source: PLoS One. 2012 Jun 29;7(6):e40012. doi: 10.1371/journal.pone.0040012 (PMC3386921; doi:10.1371/journal.pone.0040012)

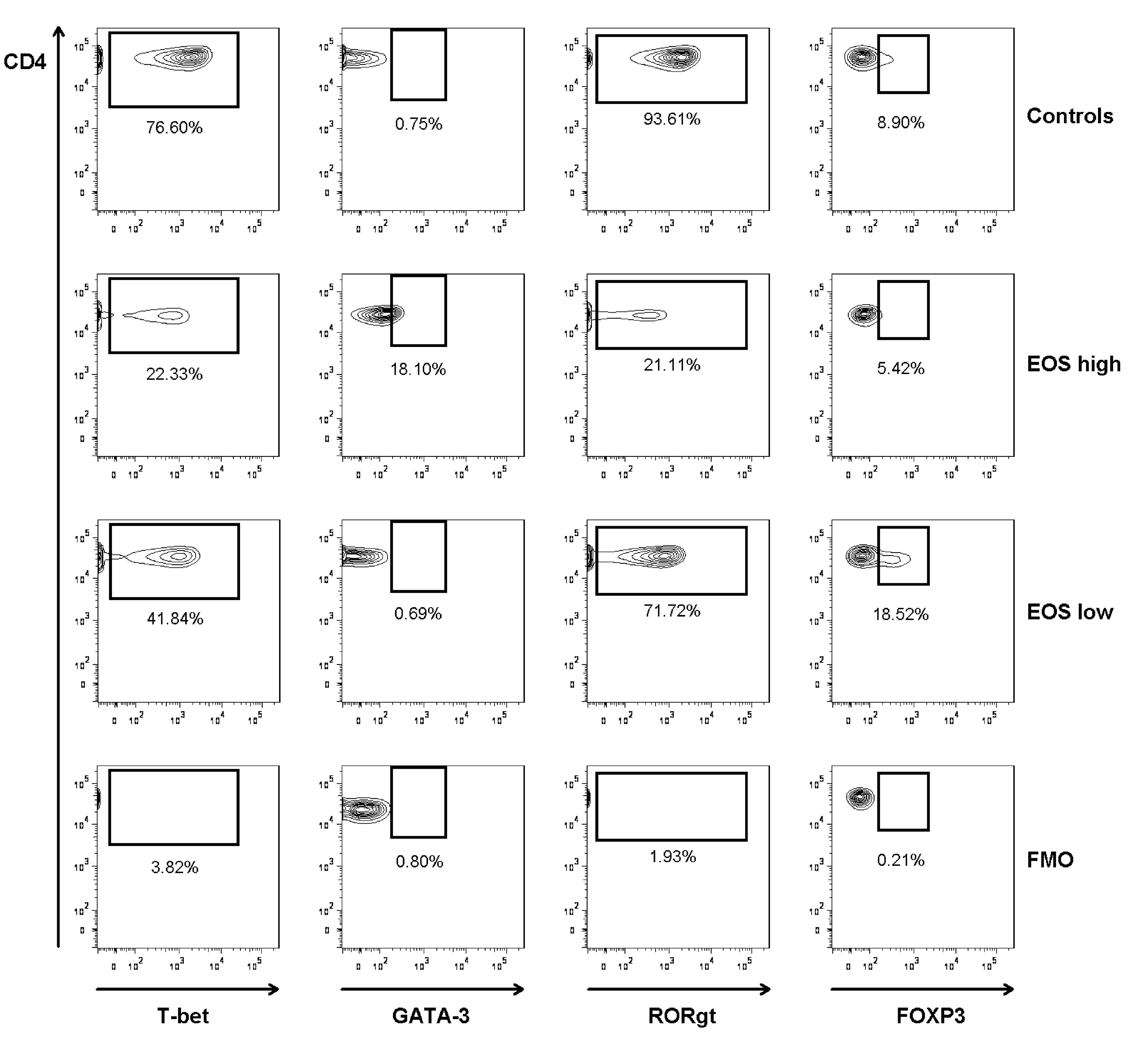

Supplement: Figure S1 — Flow cytometry analysis of Th cell subsets. Representative FACS contour plots (level 10%) showing CD4+CD25+ cells gated for T-bet (Th1), GATA-3 (Th2), RORγt (Th17) and FOXP3 (Treg). One individual from each group is presented in the top three rows. In the final row, the fluorescence minus one (FMO) control used for analyses is shown. (TIF) [file pone.0040012.s001.tif]

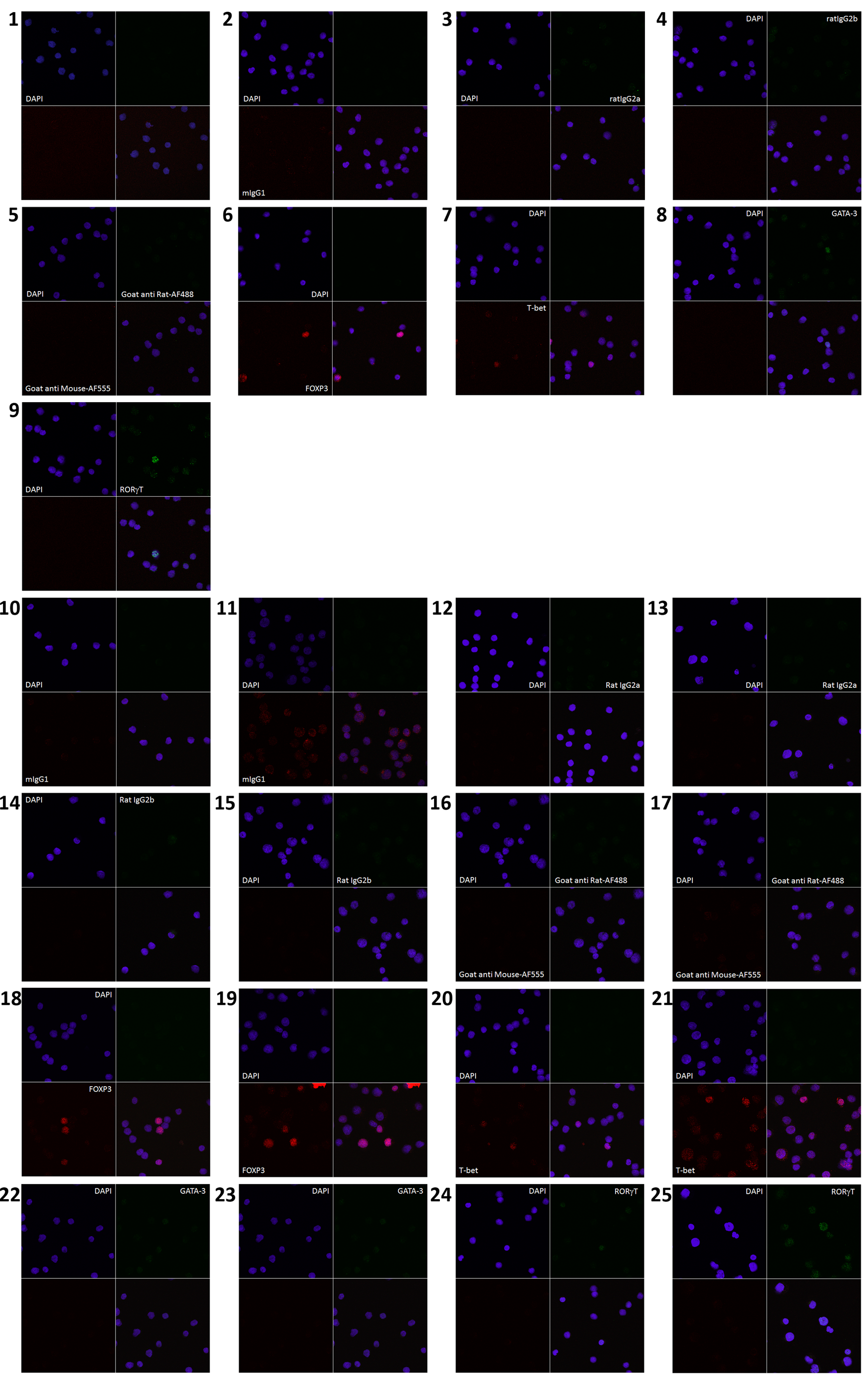

Supplement: Figure S2 — Technical controls for confocal microscopy. Z sectioning is not performed on the following samples. Micrographs of ex vivo samples: 1) Only DAPI staining; 2) DAPI/mIgG1isotype control (IC)/Goat anti-mouse IgG-AF555; 3) DAPI/ratIgG2a IC/Goat anti-rat IgG-AF488; 4) DAPI/ratIgG2b IC/Goat anti-rat IgG-AF488; 5) DAPI/No primary antibody/Goat anti-mouse IgG-AF555/Goat anti-rat IgG-AF488; 6) DAPI/mouse anti-human FOXP3/Goat anti-mouse IgG-AF555; 7) DAPI/mouse anti-human T-bet/Goat anti-mouse IgG-AF555; 8) DAPI/Rat anti-human GATA-3/Goat anti-rat IgG-AF488; 9) DAPI/Rat anti-human RORγt/Goat anti-rat IgG-AF488; Micrographs of in vitro samples: 10) DAPI/mIgG1IC/Goat anti-mouse IgG-AF555 (medium alone); 11) DAPI/mIgG1IC/Goat anti-mouse IgG-AF555 (stimulated); 12) DAPI/ratIgG2a IC/Goat anti-rat IgG-AF488 (medium); 13) DAPI/ratIgG2a IC/Goat anti-rat IgG-AF488 (stim.); 14) DAPI/ratIgG2b IC/Goat anti-rat IgG-AF488 (medium); 15) DAPI/ratIgG2b IC/Goat anti-rat IgG-AF488 (stim.); 16) DAPI/No primary antibody/Goat anti-mouse IgG-AF555/Goat anti-rat IgG-AF488 (medium); 17) DAPI/No primary antibody/Goat anti-mouse IgG-AF555/Goat anti-rat IgG-AF488 (stim.); 18) DAPI/mouse anti-human FOXP3/Goat anti-mouse IgG-AF555 (medium); 19) DAPI/mouse anti-human FOXP3/Goat anti-mouse IgG-AF555 (stim.); 20) DAPI/mouse anti-human T-bet/Goat anti-mouse IgG-AF555 (medium); 21) DAPI/mouse anti-human T-bet/Goat anti-mouse IgG-AF555 (stim.); 22) DAPI/Rat anti-human GATA-3/Goat anti-rat IgG-AF488 (medium); 23) DAPI/Rat anti-human GATA-3/Goat anti-rat IgG-AF488 (stim.); 24) DAPI/Rat anti-human RORγt/Goat anti-rat IgG-AF488 (medium); 25) DAPI/Rat anti-human RORγt/Goat anti-rat IgG-AF488 (stim.). (TIF) [file pone.0040012.s002.tif]
