# Supplementary material for: Immunophenotyping of Circulating T Helper Cells Argues for Multiple Functions and Plasticity of T Cells In Vivo in Humans - Possible Role in Asthma
Source: PLoS One. 2012 Jun 29;7(6):e40012. doi: 10.1371/journal.pone.0040012 (PMC3386921; doi:10.1371/journal.pone.0040012)
Supplement: Table S1 — Experimental laboratory data. (DOCX) [file pone.0040012.s003.docx]

**Table S1. Experimental laboratory data**

| **Parameter** | **Units** | **Healthy controls** | **Asthmatics EOS High** | **Asthmatics EOS Low** |
| --- | --- | --- | --- | --- |
|  |  | mean (±SEM) | mean (±SEM) | mean (±SEM) |
|  |  | n=9 | n=11 | n=12 |
| **Leukocytes** |  |  |  |  |
| CD45^+^ | x10^6^/L | 7138.0 (615.1) | 7564.0 (562.1) | 6611.0 (476.8) |
| **T cell subsets** |  |  |  |  |
| CD45^+^CD3^+^ | x10^6^/L | 1709.0 (221.6) | 1935.0 (183.4) | 1558.0 (109.5) |
| CD45^+^CD3^+^CD4^+^ | x10^6^/L | 1011.0 (118.0) | 1210.0 (126.8) | 950.5 (72.22) |
| CD8^+^ | x10^6^/L | 594.8 (180.0); n=5 | 790.7 (269.6); n=7 | 373.3 (50.4); n=6 |
| Ratio CD4^+^/CD8^+^ |  | 2.5 (0.6); n=5 | 2.4 (0.4); n=7 | 2.8 (0.6); n=6 |
| CD3^+^CD4^+^CD25^+^ | x10^6^/L | 622.6 (166.2) | 558.4 (169.4) | 402.3 (93.8) |
| CD3^+^TCRαβ^+^ | x10^6^/L | 1807.4 (327.1); n=5 | 2001.6 (226.6)*; n=7 | 1246.3 (157.4); n=6 |
| CD3^+^TCRγδ^+^ | x10^6^/L | 92.8 (33.0); n=5 | 68.3 (21.7); n=7 | 74.7 (21.3); n=6 |
| **Naïve and memory T cell subsets** |  |  |  |  |
| CD3^+^CD45RA^+^CD45RO^+^ | x10^6^/L | 351.8 (141.0); n=5 | 293.4 (107.0); n=7 | 147.0 (25.4); n=6 |
| CD3^+^CD45RO^+^ | x10^6^/L | 810.8 (178.5); n=5 | 937.3 (181.6); n=7 | 597.7 (63.9); n=6 |
| CD3^+^CD45RA^+^ | x10^6^/L | 751.6 (162.7); n=5 | 833.0 (90.3); n=7 | 685.0 (83.1); n=6 |
| CD3^+^CD4^+^CD45RA^+^CD45RO^+^ | x10^6^/L | 276.6 (117.8); n=5 | 206.4 (101.4); n=7 | 72.2 (18.6); n=6 |
| CD3^+^CD4^+^CD45RO^+^ | x10^6^/L | 541.8 (134.5); n=5 | 660.9 (129.4); n=7 | 444.3 (59.8); n=6 |
| CD3^+^CD4^+^CD45RA^+^ | x10^6^/L | 286.8 (96.2); n=5 | 455.3 (75.5); n=7 | 409.0 (78.6); n=6 |
| CD3^+^CD8^+^CD45RA^+^CD45RO^+^ | x10^6^/L | 74.6 (38.7); n=5 | 162.1 (108.6); n=7 | 56.5 (32.5); n=6 |
| CD3^+^CD8^+^CD45RO^+^ | x10^6^/L | 164.0 (33.3); n=5 | 286.4 (73.6); n=7 | 106.8 (12.2); n=6 |
| CD3^+^CD8^+^CD45RA^+^ | x10^6^/L | 351.4 (112.6); n=5 | 348.7 (98.3); n=7 | 219.5 (30.2); n=6 |
| **Monocytes** |  |  |  |  |
| CD14^+^ | x10^6^/L | 351.1 (49.9) | 324.2 (49.5) | 321.3 (20.76) |
| **NK cells** |  |  |  |  |
| CD16^+^CD56^+^CD3^-^CD14^-^ | x10^6^/L | 284.8 (121.0); n=5 | 310.0 (107.4); n=7 | 278.0 (49.5); n=6 |
| **B cells** |  |  |  |  |
| CD19^+^ | x10^6^/L | 272.6 (29.4) | 315.0 (33.6) | 284.1 (28.8) |

*Statistically significant in comparison with EOS low (p<0.05)
