# Supplementary material for: Immunophenotyping of Circulating T Helper Cells Argues for Multiple Functions and Plasticity of T Cells In Vivo in Humans - Possible Role in Asthma
Source: PLoS One. 2012 Jun 29;7(6):e40012. doi: 10.1371/journal.pone.0040012 (PMC3386921; doi:10.1371/journal.pone.0040012)
Supplement: Table S2 — Fold change expression of in vitro transcription factors. (DOCX) [file pone.0040012.s004.docx]

**Table S2. Fold change expression of *in vitro* transcription factors**

| **Parameter** | **Healthy**  **controls** | **Asthmatics**  **EOS High** | **Asthmatics**  **EOS Low** |
| --- | --- | --- | --- |
|  | Mean (±SEM) | Mean (±SEM) | Mean (±SEM) |
|  | n=5 | n=7 | n=6 |
|  | Fold change† | Fold change† | Fold change† |
| **T-bet^+^** GATA-3**^-^** RORγt**^-^** FOXP3**^-^** | 4.34 (3.00) | 2.45 (0.50) | 3.34 (1.05) |
| T-bet**^-^** **GATA-3^+^** RORγt**^-^** FOXP3**^-^** | 5.58 (2.03) | 13.17 (1.96)* | 12.27 (2.58) |
| T-bet**^-^** GATA-3**^-^** **RORγt^+^** FOXP3**^-^** | 10.53 (3.44) | 7.07 (1.69) | 11.50 (2.04) |
| T-bet**^-^** GATA-3**^-^** RORγt**^-^ FOXP3^+^** | 0.98 (0.09) | 1.01 (0.28) | 0.74 (0.20) |
| **T-bet^+^ GATA-3^+^** RORγt**^-^** FOXP3**^-^** | 6.04 (0.71) | 23.29 (6.32)* | 17.88 (4.43)* |
| **T-bet^+^** GATA-3**^-^** **RORγt^+^** FOXP3**^-^** | 25.47 (6.99) | 13.47 (3.36) | 20.75 (6.54) |
| **T-bet^+^** GATA-3**^-^** RORγt**^-^ FOXP3^+^** | 3.89 (1.95) | 3.08 (1.36) | 2.12 (0.93) |
| T-bet**^-^** **GATA-3^+^** **RORγt^+^** FOXP3**^-^** | 33.78 (11.18) | 35.81 (6.04) | 49.14 (11.59) |
| T-bet**^-^** **GATA-3^+^** RORγt**^-^ FOXP3^+^** | 3.15 (0.94) | 3.69 (1.44) | 2.99 (0.73) |
| T-bet**^-^** GATA-3**^-^** **RORγt^+^ FOXP3^+^** | 2.18 (0.54) | 2.64 (0.58) | 3.16 (0.75) |
| **T-bet^+^ GATA-3^+^ RORγt^+^** FOXP3**^-^** | 84.04 (20.42) | 166.06 (48.08) | 190.40 (64.80) |
| **T-bet^+^ GATA-3^+^** RORγt**^-^ FOXP3^+^** | 8.91 (4.31) | 3.97 (1.54) | 9.04 (3.35) |
| **T-bet^+^** GATA-3**^-^** **RORγt^+^ FOXP3^+^** | 7.80 (1.64) | 8.11 (2.09) | 8.52 (1.47) |
| T-bet**^-^** **GATA-3^+^ RORγt^+^ FOXP3^+^** | 18.51 (6.60) | 13.60 (1.60)** | 26.85 (4.30) |
| **T-bet^+^ GATA-3^+^ RORγt^+^ FOXP3^+^** | 49.58 (13.61) | 70.99 (14.59) | 91.46 (21.23) |

†Fold change equals Stimulated/Medium.

*Increased in comparison to control group (P<0.05).

** Decreased in comparison to EOS Low group (P<0.05).

Bold lettering highlights the transcription factor of interest.
