# Supplementary material for: Immunophenotyping of Circulating T Helper Cells Argues for Multiple Functions and Plasticity of T Cells In Vivo in Humans - Possible Role in Asthma
Source: PLoS One. 2012 Jun 29;7(6):e40012. doi: 10.1371/journal.pone.0040012 (PMC3386921; doi:10.1371/journal.pone.0040012)
Supplement: Table S3 — Antibodies used in the study. (DOC) [file pone.0040012.s005.doc]

**Table S3. Antibodies used in the study**

| **Fluorochrome conjugated antibodies** | **Clone** | **Company** |
| --- | --- | --- |
| CD3/APC-H7 | SK7 | BD Pharmingen™, BD Biosciences |
| CD4/PE-AF610 | S 3.5 | Invitrogen™, Camarillo, CA |
| CD4/APC-H7 | SK3 | BD Biosciences, San Jose, CA |
| CD8/PerCP-Cy5.5 | RPA-T8 | BD Pharmingen™, BD Biosciences |
| CD14/PE-Cy7 | M5E2 | BD Pharmingen™, BD Biosciences |
| CD14/ PerCP-Cy5.5 | M5E2 | BD Pharmingen™, BD Biosciences |
| CD16/PE | 3G8 | BD Pharmingen™, BD Biosciences |
| CD19/PerCP-Cy5.5 | SJ25C1 | BD Biosciences, San Jose, CA |
| CD19/APC | SJ25C1 | BD Biosciences, San Jose, CA |
| CD25/PE-Cy7 | M-A251 | BD Pharmingen™, BD Biosciences |
| CD45/FITC | 2D1 | BD Pharmingen™, BD Biosciences |
| CD45RA/PE-Cy7 | L48 | BD Biosciences, San Jose, CA |
| CD45RO/APC | UCHL-1 | BD Pharmingen™, BD Biosciences |
| CD56/PE | N-CAM | BD Pharmingen™, BD Biosciences |
| TCR αβ/FITC | WT31 | BD Biosciences, San Jose, CA |
| TCR γδ/PE | 11F2 | BD Biosciences, San Jose, CA |
| T-Bet/PerCP-Cy5.5 | eBio4B10 | eBioscience, San Diego, CA |
| GATA-3/PE | TWAJ | eBioscience, San Diego, CA |
| RORγt/APC | AFKJS-9 | eBioscience, San Diego, CA |
| FOXP3/ Alexa Fluor® 488 | 236A/E7 | eBioscience, San Diego, CA |
| FOXP3/PE | 236A/E7 | eBioscience, San Diego, CA |
| Ki-67/FITC | B56 | BD Biosciences, San Jose, CA |
| **Purified antibodies** |  |  |
| GATA-3 | TWAJ | eBioscience, San Diego, CA |
| FOXP3 | 236A/E7 | eBioscience, San Diego, CA |
| RORγt | AFKJS-9 | eBioscience, San Diego, CA |
| T-Bet | eBio4B10 | eBioscience, San Diego, CA |
| CD3 NA/LE | HIT3a | BD Pharmingen™, BD Biosciences |
| CD28 NA/LE | CD28.2 | BD Pharmingen™, BD Biosciences |
| **Secondary antibodies** |  |  |
| F(ab’)_2_ Goat anti mouse IgG/Alexa Fluor® 555 |  | Invitrogen™, Molecular Probes®, Eugene, OR, USA |
| Goat anti Rat IgG/ Alexa Fluor®488 |  | Invitrogen™, Molecular Probes®, Eugene, OR, USA |
