# Supplementary material for: Immunophenotyping of Circulating T Helper Cells Argues for Multiple Functions and Plasticity of T Cells In Vivo in Humans - Possible Role in Asthma
Source: PLoS One. 2012 Jun 29;7(6):e40012. doi: 10.1371/journal.pone.0040012 (PMC3386921; doi:10.1371/journal.pone.0040012)
Supplement: Methods S1 — Sputum induction and processing. (DOC) [file pone.0040012.s006.doc]

**Methods S1.**

**Sputum induction and processing**

Sputum induction and processing of whole sputum was performed according to the ERS guidelines [1] with minor modifications.

Detailed information and clear instructions were given to the participants. Measurement of pre-bronchodilator FEV_1_ was performed to ensure that participant could perform the test safely. Administration of inhaled salbutamol 0.4mgx1 was followed after 10 min by a measure of post-bronchodilator FEV_1_. If FEV_1_ was less than 1.5L or 50% of expected value the induced sputum procedure was not performed.

Induced sputum was performed by inhalation of a fixed concentration of 4% sterile saline solution for 7 min using an ultrasonic nebuliser. The nebulization was stopped if symptoms of obstruction occurred and the participant received a bronchodilator if needed. The specimen was obtained by coughing and spitting followed by a FEV_1_ measure. In the case of a fall of FEV_1_ of >20% compared with the post-bronchodilator value the test was stopped and if needed the participant received a bronchodilator. If FEV_1_<20%, the procedure was repeated.

The specimen consisting of sputum and saliva were kept cold and examined within 2h, using a modified method described by Pizzichini et al [2]. Sputum plugs were selected using a microscope, weighed and then mixed with 4 volumes x sputum weight of Sputolysin (6.5 mM dithiothreitol in 100 mM phosphate buffer, pH 7.0; Calbiochem-Novabiochem, Darmstadt, Germany). PBS was added in an equal volume to the homogenized sputum and then passed through a 70 μm cell strainer (BD Biosciences Europe, Heidelberg, Germany). The cell suspension was centrifuged and the cell pellet was resuspended in PBS. Cytospins were prepared from the cell pellet and differential cell counts were established by counting 500 cells. The results of the differential counts are expressed as a percentage of the total number of non-squamous cells.

**References**

1. Paggiaro PL, Chanez P, Holz O, Ind PW, Djukanovic R, et al. (2002) Sputum induction. Eur Respir J Suppl 37: 3s-8s.

2. Pizzichini E, Pizzichini MM, Efthimiadis A, Evans S, Morris MM, et al. (1996) Indices of airway inflammation in induced sputum: reproducibility and validity of cell and fluid-phase measurements. Am J Respir Crit Care Med 154: 308-317.
